# Immune Epigenetic Crosstalk Between Malignant B Cells and the Tumor Microenvironment in B Cell Lymphoma

**DOI:** 10.3389/fgene.2022.826594

**Published:** 2022-02-14

**Authors:** Patrizia Mondello, Stephen M. Ansell, Grzegorz S. Nowakowski

**Affiliations:** Division of Hematology, Mayo Clinic, Rochester, MN, United States

**Keywords:** epigenetics, tumor microenvironment, immune crosstalk, B cell lymphoma, T cells, diffuse large B cell lymphoma, follicular lymphoma

## Abstract

Epigenetic reprogramming is a hallmark of lymphomagenesis, however its role in reshaping the tumor microenvironment is still not well understood. Here we review the most common chromatin modifier mutations in B cell lymphoma and their effect on B cells as well as on T cell landscape. We will also discuss precision therapy strategies to reverse their aberrant signaling by targeting mutated proteins or counterbalance epigenetic mechanisms.

## Introduction

Among the highly recurrent mutations in diffuse large B cell lymphoma (DLBCL) and follicular lymphoma (FL), almost 60% occur in transcription factors or epigenetic modifier proteins (Morin et al., 2011; Reddy et al., 2017; Chapuy et al., 2018; Schmitz et al., 2018). While mutations in chromatin modifier genes (CMGs) have been associated with lymphomagenesis, their role in reshaping the tumor microenvironment (TME) remains poorly characterized. Emerging evidence suggests that malignant B cells may influence the surrounding immune composition through a direct reprogramming of their immune receptors, which in turn disrupt the immune synapse between B and T cells, and indirectly by releasing of cytokines that impact immune response.

The notion of such an epigenetic circuit is plausible in the context of the germinal center (GC) B cells from which DLBCL and FL arise. Epigenetic mechanisms play a critical role in the entry and exit of B cells to the GC. Normally, antigen presentation and BCR signaling are attenuated in the GC dark zone (DZ) to enable B cells to freely proliferate and undergo somatic hypermutation. However, signals received by the B cells in the GC light zone (such as CD40L) induce expression of antigen presentation genes, so that high affinity B cells can be selected to exit the GC reaction and undergo terminal differentiation to plasma cells(Mesin et al., 2016). During selection, a fundamental role is played by T follicular helper (T_FH_) CD4^+^ cells - characterized by the expression of CXCR5+, PD1+ and BCL6+ - and follicular dendritic cells (FDCs), which engage GC B cells to promote clonal selection and affinity maturation(Crotty, 2014). In contrast, suppressive CD4^+^ FOXP3+ T regulatory (T_reg_) cells, called T follicular regulatory (T_FR_) cells, limit the function and output of GC reaction (Chung et al., 2011; Wollenberg et al., 2011). While T_FR_ cells dominate the early-GC (Wing et al., 2017), a distinct T_reg_ population prevails in the late-GC (Jacobsen et al., 2021). These recently described cells seem to arise from T_FH_ cells, however it remains unclear what triggers their acquisition of regulatory properties (Jacobsen et al., 2021).

**FIGURE 1:**
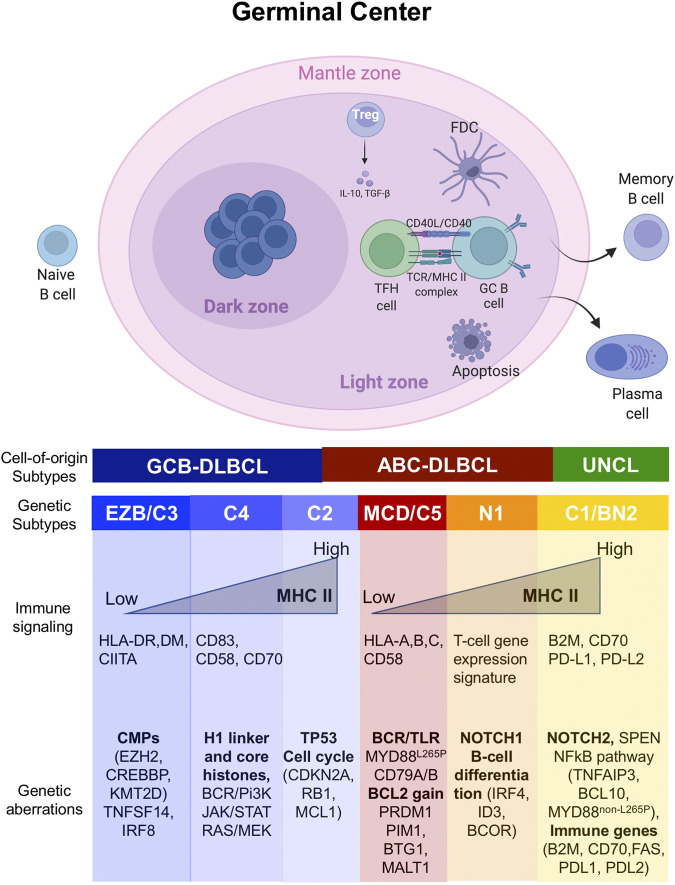
Genetic lesions associated with distinct transcription and molecular subtypes of DLBCL. Schematic representation of the germinal center (GC) reaction and its relationship with the major molecular and genetic subtypes of DLBCL. The GC forms upon encounter of a naïve B cell with an antigen, and it is divided in two distinct compartments: the dark zone (DZ) and the light zone (LZ). In the GC DZ, B cells freely proliferate and undergo somatic hypermutation. However, signals from the GC LZ induce expression of antigen presentation in B cells, so that high affinity B cells can be selected to exit the GC reaction and terminally differentiate into plasma cells or memory B cells. Cells that are not selected undergo apoptosis. As the B cells repeatedly cycle between DZ and LZ, they might acquire mutations in genes predominantly activated in the different GC phases, translating in a distinct genetic, transcriptional and phenotypic subtype which might shape the surrounding tumor microenvironment.

**FIGURE 2:**
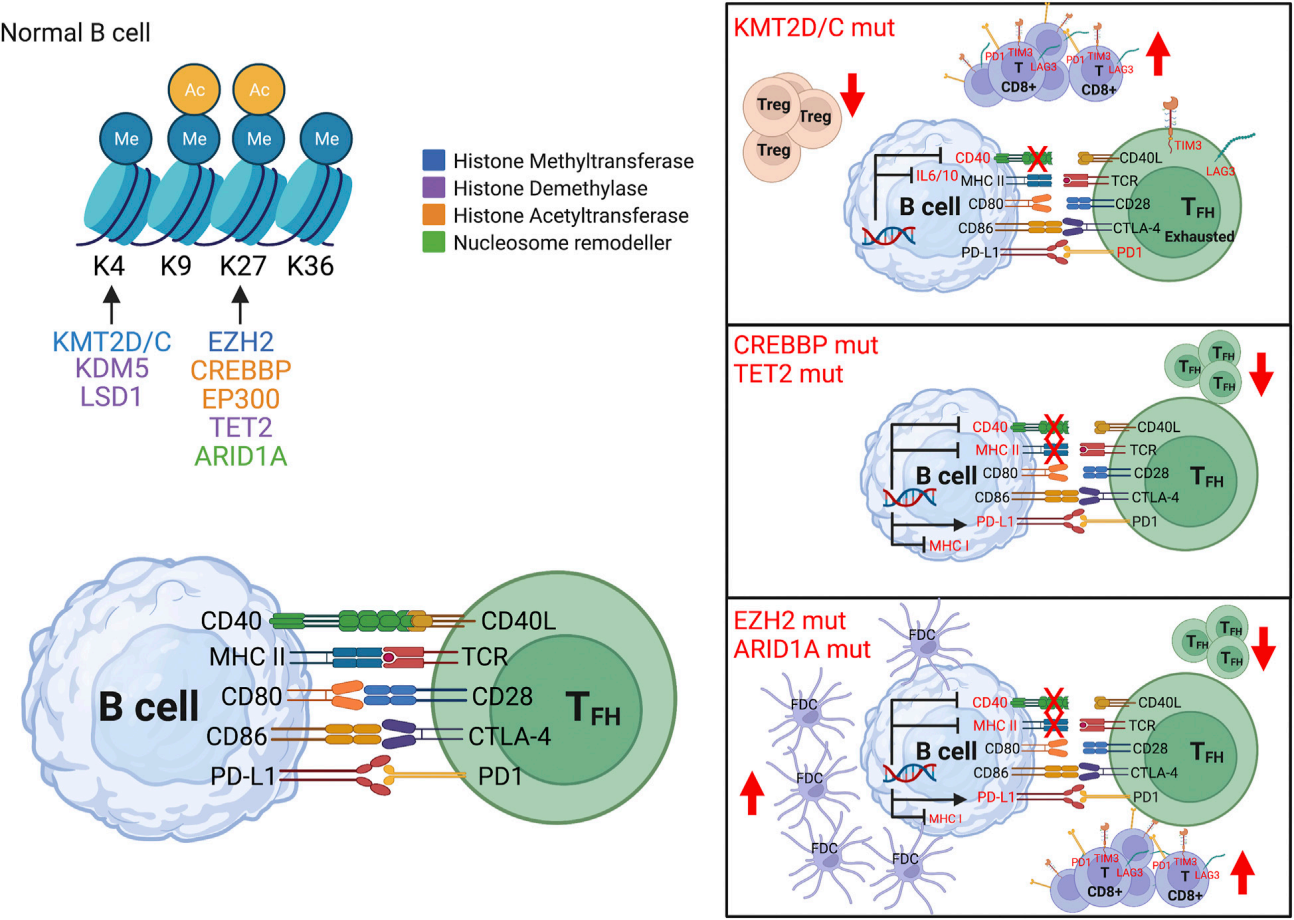
Epigenetic control on immune signaling. In normal B cells, epigenetic mechanisms (DNA methylation, histone modification and chromatin remodeling) control gene expression including immune signaling (left top panel), and in turn modulate immune receptors expression (left bottom panel). In B cell lymphoma, aberrant epigenetic programming can disrupt the immune synapse between B and T cells with concordant reshaping of the immune microenvironment. The most frequent epigenetic alterations are: 1) Mutations in KMT2D/C (right top panel) which suppress CD40, IL10-IL6, NF-kB signaling, with prevalence of exhausted CD8^+^ T cells and decrease of T regulatory (T_reg_) cells. 2) Mutations in CREBBP and TET2 (right middle panel) which repress antigen presentation genes (such as MHC class II, its transcription factor CIITA), increase inhibitory molecules (such as PDL1), with lower infiltration of CD4^+^ T cells. 3) Mutations in EZH2 and ARID1A (right lower panel) which repress CIITA (master regulator of MHC class II), NLRC5 (transactivator of MHC class I) and CD40, with decrease in T follicular helper (T_FH_) cells and increase in follicular dendritic cells (FDC).

Somatic mutations of CMGs disrupt the fine-tuned mechanism governing GC (Figure 1), thus promoting lymphomagenesis and reprograming TME toward immune suppressive lymphoma niche (Ortega-Molina et al., 2015; Boice et al., 2016; Ennishi et al., 2019; Mondello et al., 2020; Krull et al., 2021; Amin and Braza, 2022). Despite retaining the characteristics of the primary site of development, the cellular composition and spatial arrangement of the TME mirror the genetic complexity and tumor type (Scott and Gascoyne, 2014). Accordingly, the TME has shown a close association with treatment failure and outcome (Dave et al., 2004; Kotlov et al., 2021; Mondello et al., 2021). Here we provide an overview of the most frequent epigenetic mechanisms associated with B cell lymphoma (Figure 1, 2 and Table 1) and discuss the therapeutic options to revert their oncogenic and immunosuppressive effect.

**TABLE 1:** The most common mutations of epigenetic factors and their effect on the tumor microenvironment.

Epigenetic modifiers	Function	Mutation frequency	Affected genes linked to immune signaling	Changes in TME
KMT2D	H3K4 methyltransferase	30% DLBCL	CD40, IL-10-IL6	Increase of exhausted CD8^+^ T cells Brisou el al. (2018) and of effector immune cells Wang et al. (2020)
		80% FL		Decrease in T_reg_ cells Wang et al. (2020)
KMT2C	H3K4 methyltransferase	5% DLBCL	IL1, GPX8, GSTA4, GSSTT1, ETS/PU.1, IRF, RUNX, AP-1	Increase of CD8^+^ T cells and of effector immune cells Liu et al. (2021); Zhang et al. (2021)
		13% FL		Decrease in T_reg_ cells Liu et al. (2021); Zhang et al. (2021)
CREBBP	Lysine acetyltransferase	15% DLBCL	MHC II, CIITA, CD74, CD40, PDL1, IFN	Decrease CD4^+^ T cells Green el al. (2015)
		60% FL		
EP300	Lysine acetyltransferase	5% DLBCL	E2F1, CDC25B, CDKN1A, PDNA, CDT1, DNA2, RADE51, XRCC1	Increase of M2 macrophages Huang el al. (2021)
		15% FL		Decrease in T_reg_ cells Liu et al. (2013)
EZH2	H3K27 methyltransferase	25% DLBCL	CIITA, NLRC5, Th1-type chemokines	Increase in FDCs Béguelin et al. (2020)
		10–25% FL		Decrease in T_FH_ cells Béguelin et al. (2020)
TET2	Methylcytosine dioxygenase 2	10% DLBCL	CD40, IL-10-IL6, MHC II	Increase of CD8^+^ T cells memory Tsagaratou et al. (2017) and NK cells Carty et al. (2018)
		2% FL		Decrease T cells Pan et al. (2017) and macrophages Lee el al., 2021
ARID1A	SWI/SNF component	10% DLBCL	IFN, PDL1	Increase of CD8^+^ T cells Shen et al. (2018)
		11% FL		
HIST1H1C/E	Linker histone	26% DLBCL	NANOG, SOX2, PRC2	Not yet explored
		44% FL		

## KMT2D

The *KMT2D* gene (also known as MLL2 or MLL4) encodes a SET domain-containing lysine methyltransferase and is one of the most frequently mutated genes in B cell lymphomas, reported as being affected in up to 30% of DLBCL and 80% of FL (Morin et al., 2011; Pasqualucci et al., 2011; Ortega-Molina et al., 2015; Zhang et al., 2015; Chapuy et al., 2018; Schmitz et al., 2018). However, KMT2D mutations have not been associated with progression-free survival (PFS) or overall survival (OS) (Ortega-Molina et al., 2015). The majority of these mutations are nonsense or frameshift events that yield truncated proteins lacking the C-terminal SET domain, resulting in enzymatic loss of function (Morin et al., 2011; Pasqualucci et al., 2011; Ortega-Molina et al., 2015; Zhang et al., 2015). Loss of *KMT2D* in B cells in mice leads to the development of B-cell lymphomas, indicating that this gene is a *bona fide* tumor suppressor (Ortega-Molina et al., 2015; Zhang et al., 2015). The normal function of *KMT2D* is linked to its mediating H3K4 mono and demethylation (H4K3me1/2) primarily at gene enhancers (Hu et al., 2013; Lee et al., 2013; Wang et al., 2016). This histone mark is required for non-coding genomic elements to manifest functionality as enhancers (Kouzarides, 2007; Li et al., 2007). *KMT2D* loss of function was shown to result in focal loss of H3K4me1/2 primarily at gene enhancers, that results in aberrant repression of genes involved in immune signaling such as CD40, IL10-IL6, and NFkB signaling (Ortega-Molina et al., 2015; Zhang et al., 2015). These gene sets play a critical role also in the terminal differentiation of B cells towards the memory or plasma cell fate. Several tumor suppressor genes such as *TNFAIP3* and *SOCS3* also become silenced upon *KMT2D* loss of function (Ortega-Molina et al., 2015). Remarkably, *KMT2D* inactivation promotes remodeling of the TME with prevalence of exhausted CD8^+^ T cells strongly co-expressing inhibitory receptors (Lag3, Tim3, Pdcd1), suggesting that loss of *KMT2D* not only reprograms B cells, but also shapes a supportive lymphoma niche which contributes to immune escape (Brisou et al., 2018) Accordingly, a pooled mutagenic screening with CRISPR-mediated genetically engineered mouse models (CRISPR-GEMM) identified *KMT2D* as a major modulator of response to immune checkpoint blockade (ICB) with increase of effector immune cells (e.g., CD8^+^ T cells, NK cells and M1 macrophages) and decrease of T_reg_ cells in the TME (Wang et al., 2020). Although *KMT2D* deficiency seems to sensitize tumors to ICB in solid cancers, this might not be the case for B cell lymphoma where concurrent mutations might alternatively lead to immune escape.

Since mutations in *KMT2D* cause loss of function and are replicated by genetic deletion of this gene, there is no immediately obvious opportunity for development of precision targeted therapies to reverse their effect. Lymphomas with *KMT2D* somatic mutations manifest reduction (but not complete loss) of enhancer H3K4 methylation (Ortega-Molina et al., 2015). The reasons for this could be simply due to loss of histone methyltransferase activity, or possibly due to the actions of putative histone demethylases that might normally counteract the actions of *KMT2D*. This latter scenario is intriguing since it would imply that *KMT2D* forms part of a reversible epigenetic circuit with histone methyltransferases and histone demethylases competing to set enhancers into a more active or repressed configuration. Therefore, it is possible that in the presence of *KMT2D* mutation a histone demethylase could act relatively unopposed to maintain silencing of the GC differentiation program. If this was the case it would follow that *KMT2D* mutant DLBCL cells would become biologically dependent on such a demethylase, which would thus represent a potential precision epigenetic therapy target for these patients. There are two families of enzymes that specifically demethylate H3K4me1/2 (Højfeldt et al., 2013). The first to be discovered were the FAD dependent amino-oxidases KDM1A (LSD1). LSD1 is highly expressed in GC B-cells and DLBCLs. However, previous work ruled out LSD1 as the KMT2D antagonist in DLBCL and FL cells, since specific inhibitors against this protein do not revert silencing of KMT2D target genes nor cause any kind of proliferation arrest or differentiation in DLBCL cells (Hatzi et al., 2019). The second family of H3K4 demethylases are the KDM5 family of jumonji proteins consisting of KDM5A (JARID1A/RBP2), KDM5B (JARID1B/PLU1), KDM5C (JARID1C/SMCX) and KDM5D (JARID1D/SMCY). Recently, inhibition of the demethylases KDM5 (KDM5i) has showed to increase H3K4me3, restore the gene expression repressed on loss of *KMT2D* and cause antiproliferative effect *in vitro* and *in vivo*. (Targeting KDM5 demethylases counteracts KMT2D loss of function in diffuse large B-cell lymphoma; Heward et al., 2021) Additionally, reactivation of silenced CD40 pathway genes by KDM5i yields a powerful synthetic lethal effect in combination with CD40 agonist antibodies (Targeting KDM5 demethylases counteracts KMT2D loss of function in diffuse large B-cell lymphoma), which can open the opportunity for novel combination targeted therapies.

## KMT2C

The *KMT2C* gene (also known as MLL3) is another of the four members in the mixed lineage leukemia (MLL) family. It also encodes a histone methyltransferase that mediates mono- and tri-methylation of histone H3 at lysine K4 (H3K4me1 and H3K4me3), suggesting a partial redundancy with KMT2D in lymphomagenesis (Lee et al., 2013). However, mutations in *KMT2C* are found at a much lower frequency with 5–13% incidence in DLBCL and FL, respectively (Reddy et al., 2017; Chapuy et al., 2018; Schmitz et al., 2018). The *KMT2C* mutations are not mutually exclusive with those involving *KMT2D*, suggesting that their function may not completely overlap. Nevertheless, their redundancy remains a matter of debate due to the contrasting results on the effect of *KMT2D* on genome-wide distribution of H3K4 methylation (Ortega-Molina et al., 2015; Zhang et al., 2015). Therefore, future studies are required to clarify the relation between these two enzymes in GC B cells and in lymphomagenesis. Interestingly, *KMT2C* mutations showed a positive association with tumor infiltration of CD8^+^ T cells, M1 macrophages, neutrophils and NK cells, while they negatively correlated with T_reg_ cells, and in turn predicted response ICB and favorable outcome (Liu et al., 2021; Zhang et al., 2021). However, the putative immune signaling responsible for the remodeling of the TME remains elusive.

## CREBBP

Approximately 30–40% of DLBCL and FL manifest somatic mutations of the *CREBBP* histone acetyltransferase (Morin et al., 2011; Pasqualucci et al., 2011). These occur early during pathogenesis and are more frequent in GCB DLBCL (Green et al., 2015; Jiang et al., 2017). Inactivating missense mutation of the histone acetyltransferase (HAT) domain account for 50–70% of cases, whereas most remaining alleles cause truncation or loss of expression (Morin et al., 2011; Pasqualucci et al., 2011). CREBBP mutations have been associated with adverse outcome in FL (Pastore et al., 2015; Mondello et al., 2020) and DLBCL (Huang et al., 2021), however when discriminating point mutations from nonsense/frameshift mutations only failure-free survival remained significant, but not OS (Mondello et al., 2020). The normal function of *CREBBP* is to mediate H3K27 acetylation at specific gene enhancers required for terminal differentiation and immune signaling. In the GC dark zone these enhancers are temporarily repressed by the BCL6/SMRT/HDAC3 complexes through H3K27 deacetylation (Hatzi et al., 2013). Then, signals for the GC light zone dissociates HDAC3 from BCL6, and drives CREBBP to “toggle” these enhancers back to the active state by restoring H3K27 acetylation (Hatzi et al., 2013; Jiang et al., 2017), which induces expression of antigen presentation genes (such as MHC class II, its transcription factor CIITA, and its co-receptor CD74), so that high affinity B-cells can be selected to exit the GC reaction.

*CREBBP* mutation induces focal loss of enhancer H3K27 acetylation and concordant repression of antigen presentation, CD40 and BCR signaling genes. As a consequence, *CREBBP* mutant FLs featured lower infiltration of CD4^+^ T cells and were impaired in activating autologous T cells as compared to CREBBP wild type (WT) lymphoma *ex vivo* (Green et al., 2015; Jiang et al., 2017). This is the result of impaired acetyltransferase activity and unopposed deacetylation by BCL6/SMRT/HDAC3 complexes at enhancer of B cell signaling and immune response genes (Jiang et al., 2017). Hence, lymphomas with *CREBBP* mutation become extremely dependent on HDAC3. Similarly, CREBBP knockout downregulated MHC class II (García-Ramírez et al., 2017; Hashwah et al., 2017) while MHC class II deletion phenocopies the effect of CREBBP knockout and CD4^+^ depletion on lymphomagenesis (Hashwah et al., 2017). These data support the notion that immune evasion is the key effect of CREBBP mutation. Recently, HDAC3 inhibition showed to counteract aberrant CREBBP by reverting the unopposed deacetylation of BCL6/SMRT/HDAC3 complexes on enhancers genes involved in antigen presentation and terminal differentiation. Targeting HDAC3 caused more profound growth suppression and cell death in *CREBBP* mutant than WT DLBCL cell lines (Mondello et al., 2020). Reciprocally, conditional deletion of *HDAC3* in GC B cells impaired GC formation and induced upregulation of genes repressed in *CREBBP* mutant patients (Jiang et al., 2017). Furthermore, selective HDAC3 inhibitors restored immune surveillance by reactivating BCL6-repressed INF pathway and antigen presentation genes in lymphoma cells, enabling T-cells to recognize and kill them, especially in the presence of CREBBP mutation (Mondello et al., 2020). As HDCA3 is a crucial repressor of *PDL1* transcription (Deng et al., 2019), HDAC3 inhibition increases PDL1 expression on B cells (Deng et al., 2019; Mondello et al., 2020). Thus, combining HDAC3 inhibitors with PDL1 checkpoint blockade enhances tumor regression through direct cell autonomous activity and enhanced antitumor immune response(Mondello et al., 2020).

## EP300

EP300 is a paralog of CREBBP gene and similarly encodes for histone acetyltransferase. However, it is found mutated only in 5–15% of DLBCL and FL and largely in mutually exclusivity with CREBBP (Morin et al., 2011; Pasqualucci et al., 2011; Chapuy et al., 2018; Schmitz et al., 2018). Interestingly, EP300 and CREBBP display structural and functional similarity with 60% shared amino acid identity (Chan and La Thangue, 2001), suggesting their potential compensatory function. However, despite having common transcriptional signatures, they also present distinct transcriptional targets in GC B cells (Meyer et al., 2019). Specifically, mice with conditional deletion of EP300 showed down-regulation for genes involved in cell cycle (e.g. E2F1, CDC25B, CDKN1A), DNA replication (PCNA, CDT1, DNA2) and DNA repair (RAD51 and XRCC1) while those deficient for CREBBP were reduced for genes involved in antigen presentation (e.g., CIITA, H2-DM) and terminal differentiation (e.g., IRF4, SPIB, NFKB2 and CD40). The difference in dysregulated transcriptional programs is reflection of the two distinct GC compartments largely involved - the dark zone and the light zone—whose enrichment was confirmed by gene set enrichment analysis (Meyer et al., 2019). Remarkably, combining genetic deletion of both genes prevented GC formation *in vivo* and impaired DLBCL proliferation *in vitro*, suggesting that DLBCLs with CREBBP mutations become biologically dependent on EP300 (Meyer et al., 2019). Additionally, EP300 mutation seems to increase infiltration of M2 macrophages *via* FCXW7-NOTCH-CCL2/CSF1 axis and associate with high level of the immunosuppressive cytokine IL-10 (Huang et al., 2021), while its inhibition impairs T_reg_ cells, therefore promoting anti-lymphoma immune response (Liu et al., 2013).

## EZH2

*EZH2* is a histone methyltransferase important for bivalency at key promoters that regulate the GC B cell phenotype (Velichutina et al., 2010; Béguelin et al., 2013; Caganova et al., 2013). *EZH2* mutations are present in about 10–25% of FL, 25% of GCB DLBCLs while virtually absent in the ABC subtype (Morin et al., 2011; Béguelin et al., 2013). Most frequently the mutations are gain-of-function events that occur in the protein’s SET domain, resulting in increased trimethylation at H3K27 compared with wild-type. *EZH2* mediates its effect by suppressing genes involved in proliferation check point, GC exit and terminal differentiation (Béguelin et al., 2013). This translates into persistent centroblast proliferation, which in presence of additional oncogenic cooperator events enable transformation(Sneeringer et al., 2010; Yap et al., 2011; McCabe et al., 2012a; Béguelin et al., 2013). Furthermore, *EZH2* mutation represses *CIITA*, which is master regulator of MHC class II, *NLRC5*, which is a transactivator of MHC class I, and Th1-type chemokines (Peng et al., 2015). The epigenetic reprogramming of GC B cells modifies the surrounding microenvironment toward immune suppression which further boosts lymphomagenesis (Beguelin et al., 2020). In Ezh2-Y641F conditional knock-in mice there was silencing of genes (*CD69, ICOSL, ICAM1, ICAM2, SLAM, LY108* and *BASP1*) involved in the immune synapse between GC B cells and T_FH_ cells with concordant disruption of CD40/CD40L signaling and of B-T cells immune module (Liu et al., 2015; Zaretsky et al., 2017; Ise et al., 2018; Beguelin et al., 2020). In the absence of T_FH_ interaction, GC persisted only in *EZH2* mutant, but not *EZH2* WT GC cells due to the dense network with FDCs which seems to play a supportive role. This network is sustained in part by the aberrant expression of genes involved in the homeostasis of FCDs, such as *TNFRSF13C* and *LTB* (Beguelin et al., 2020).

Clinically, EZH2 mutation has been associated with favorable outcome in FL patients who received R-CHOP or chemo-free regimens (Pastore et al., 2015; Huet et al., 2017; Lockmer et al., 2020), while no prognostic association was observed with those who received rituximab plus bendamustine (Jurinovic et al., 2019), suggesting a potential role for therapy decision-making. On the contrary, aberrant EZH2 seemed associated with an inferior prognosis in the novel molecular subtypes C3 (*p* = 0.05) (Chapuy et al., 2018) and EZB (*p* = 0.06) (Schmitz et al., 2018) compared to other GCB DLBCL. Nevertheless, it should be noted that the genetic clusters include other mutations which may impact on the survival. Several EZH2 inhibitors (EZH2i) arrest proliferation and induce apoptosis of *EZH2*-mutant B cells by blocking H3K27me3, resulting in the reactivation of silenced gene targets (McCabe et al., 2012b; Knutson et al., 2012; Béguelin et al., 2013). In line with these data, the clinical development of EZH2i has demonstrated a superior efficacy in FL patients with EZH2 mutations compared to those with wild-type EZH2, however a modest activity was observed also in the latter likely due to other concordant mutations that indirectly activate EZH2 and/or conserved baseline EZH2 activity (Morschhauser et al., 2020; Mondello and Ansell, 2022). Additionally, treatment with EZH2i restored antigen presentation including MHC class I and II and increased infiltration of T cells in the TME as well as the expression of immune checkpoint markers. This negative feedback opens the possibility for combination therapy with ICB (Ennishi et al., 2019).

## TET2

*TET2* encodes a dioxygenase that converts 5-methylcytosine (5mC) to 5-hydroxymethylcytosine (5hmC) (Ito et al., 2011). 5mC is required for DNA demethylation in tandem with the base excision repair machinery, however it also activates transcription independently (Cimmino et al., 2011; Wu et al., 2011; Rampal et al., 2014). Somatic mutations of *TET2* occur in about 10% of DLBCL (Asmar et al., 2013; Reddy et al., 2017) and display a tumor suppressor role (Dominguez et al., 2018). *TET2* loss leads to focal loss of enhancer hydroxymethylation and repression of genes involved in GC exit. Accordingly, a transgenic mouse model with deletion of *TET2* showed GC hyperplasia, block of plasma cell differentiation and preneoplastic phenotype (Dominguez et al., 2018). Notably, the enhancers and genes dysregulated in tumors with aberrant TET2 are the same as those repressed in *CREBBP*-mutant lymphoma. These include CD40, cytokines (IL6 and IL10) and antigen presentation gene sets (Dominguez et al., 2018). *TET2* and *CREBBP* mutations are mutually exclusive. *TET2*-dependent loss in hydromethylation impairs H3K27 acetylation, suggesting that *CREBBP* requires *TET2* and the 5hmC to mediate H3K27 acetylation. In support of this, a superior sensitivity to treatment with HDAC3 inhibitor was observed in cells with *TET2* loss (Dominguez et al., 2018). Furthermore, mouse studies have shown that *TET2* reshapes chromatin accessibility at genomic binding regions of key transcription factors involved in immune signaling (e.g., *BATF* and *ETS1*) favoring disfunction of tumor infiltrating lymphocytes (TIL) (Lee et al., 2021). Conversely, *TET2* deletion augments CD8^+^ T cell memory (Carty et al., 2018), induces cell proliferation of natural killer T cells (Tsagaratou et al., 2017) and significantly enhanced anti-tumor activity of the TIL in a similar manner as that observed with anti-PDL1 blockade, however no synergistic effect was observed combining *TET2* depletion and ICB (Lee et al., 2021). Additionally, loss of *TET2* significantly enhanced immune response to CAR T cell therapy and suppressed tumor growth (Pan et al., 2017; Fraietta et al., 2018).

## ARID1A

*ARID1A* (also known as BAF250a) is a critical component of the multi-subunit remodeling complexes SWI/SNF (SWItch/Sucrose Non-Fermentable), which control chromatin condensation and accessibility. Mutations in ARID1A are more commonly frameshift mutations and present in mutual exclusivity with its paralogous subcomponent SMARCA4. Normally, this complex modulates gene expression involving cell proliferation and differentiation (Mittal and Roberts, 2020). ARID1A can also bind directly to p53, promoting its activation as well as the expression of the cell cycle inhibitor CDKN1A (Guan et al., 2012). Additionally, ARID1A seems to be involved in FAS-mediated apoptosis, which is a critical mechanism for clonal deletion of GC B cells (Luo et al., 2008). However, the role of ARID1A in lymphomagenesis remains unclear. Mutations in *ARID1A* compromises mismatch repair resulting in increased tumor mutational load and genetic instability (Mittal and Roberts, 2020). In line with these findings, a remarkable efficacy was observed using inhibitors of the DNA damage checkpoint kinase ATR as well as PARP inhibitors in *ARID1A* mutant tumors (Shen et al., 2015). Additionally, aberrant *ARID1A* expression impairs IFN signaling, leading to immunosuppressive TME (Li et al., 2020) which is characterized by increased PDL1 expression and cytotoxic CD8^+^ T cell infiltration (Shen et al., 2018). Mechanistically, ARID1A displays an antagonistic effect with EZH2 and functionally competes for IFN signaling, which may account for immune response (Li et al., 2020). This novel mechanism ushers the opportunity for therapeutic vulnerabilities. For example, mutations of *SWI/SNF* genes have been associated with increased sensitivity to ICB (Shen et al., 2018).

## Histone 1C/E

Linker histone H1 proteins are responsible for the nucleosome structure of the chromosomal fiber in eukaryotes (Fyodorov et al., 2018). They repress transcription by limiting chromatin accessibility (Fan et al., 2005) either directly through condensation of chromatin fiber, or indirectly *via* recruitment of transcriptional repressors or impairment of transcriptional activators (Fyodorov et al., 2018). Their function is poorly understood, however *H1c/d/e* knockout mice exhibited impaired embryonic stem cell differentiation (Zhang et al., 2012), suggesting a critical role in cellular phenotyping. Histone H1 mutations are highly recurrent in B cell lymphoma, with *H1C* and *H1E* mutations being the most frequent and occurring in ∼30% of DLBCL and FL (Li et al., 2014; Okosun et al., 2014). Recently, Yusufova et al. showed how disruption of H1 proteins results in architectural remodeling with focal opening of chromatin throughout the all genome. This decompaction was associated with gain of H3K36me2 and/or loss of repressive H3K27me3, and concordant re-expression of the normally silent developmental genes. Loss of *H1c* and *H1e* in mice also associated with increased proliferation of the GC B cells, ultimately leading to an aggressive lymphoma with T cell infiltrates as reminiscence of ABC subtype of DLBCL (Yusufova et al., 2021). These data establish *H1* as a *bona fide* tumor suppressor since its aberrant expression drives lymphomagenesis through architectural reorganization of genomic compartments, which in turn leads to epigenetic reprogramming and subsequent reactivation of “stemness” genes. Future studies will be needed to explore the impact of GC B cells differently expressing H1 linkers on the immune components of the microenvironment.

## Conclusion and Perspective

Over the last 2 decades we have witnessed the breakthrough of immunotherapy. However, this therapeutic success has been damped in non-Hodgkin B cell lymphoma. Part of the reason is the presence of intrinsic and/or extrinsic immune escape strategies that lymphoma cells develop to survive. The most frequent mechanisms are 1) “hide” from the immune system through genetic or epigenetic alterations that mask molecules involved in antigen presentation or immune receptors, or 2) “defend” from immune eradication through activation of anti-apoptotic mechanisms (e.g., perforin/granzyme pathway, extrinsic pathway including FAS and TRAIL death receptors, and intrinsic pathway including BL2 family proteins) and/or expression of inhibitory molecules (PDL1/L2, CD47, FASL) and/or release of immunosuppressive cytokines (IL10, IL4, TGF-β) (de Charette and Houot, 2018). Despite the remarkable scientific improvements, we are still far from a deep understanding of the cancer immune surveillance. Particularly, the driving mechanisms that are responsible for the distinct tumor microenvironment remain unknown. Several studies have pointed towards aberrant epigenetic programming as a critical player in shaping the lymphoma immune niche. Interestingly, many of the above-mentioned CMGs control—with some degree of overlap - the same GC B cell transcriptional programs by targeting either the enhancers (CREBBP/EP300, KMT2D/KMT2C, TET2) or the promoters (EZH2, ARID1A). Similarly, these genes control a “GC B cell exit” immune module involving multiple immune recognition molecules, including MHC class II, CD40 and PDL1, as well as induce cytokines release which further influence the composition of the TME. It is possible that mutations of CMGs could promote lymphomagenesis while causing aberrant silencing of these immune signaling, which leads, for example, to disruption of the immune synapse between B and T cells, ultimately impairing the immune surveillance.

To decipher the crosstalk between B and T cells, future work must further elucidate the epigenetic circuits regulating immune signaling, and the different ways of modulating the acquisition or removal of histone markers. Additional knowledge on the role of CMGs in normal and malignant B cell and immune cells is mandatory and must be supplemented by functional evidence of a possible inter-influence. For instance, structural rearrangement of immune receptor including downregulation of stimulatory immune molecules and upregulation of inhibitory molecules in B cells could lead to decrease and/or exhaustion of T_FH_ cells. Identification of these mechanisms might be quite impactful as it would open opportunity for precision therapy with a dual mechanism of action by targeting synthetic vulnerabilities while restoring immune response. This novel therapeutic approach might also be exploited to overcome the existing limitation with cellular therapy, with the potential to yield maximal anti-tumor immunity.
